# Preparation of Novel Nanoformulation to Enhance Efficacy in the Treatment of Cardiovascular Disease

**DOI:** 10.3390/biomimetics7040189

**Published:** 2022-11-04

**Authors:** Santhoshkumar Jayakodi, Hyunjin Kim, Soumya Menon, Venkat Kumar Shanmugam, Inho Choi, Medidi Raja Sekhar, Rakesh Bhaskar, Sung Soo Han

**Affiliations:** 1 Department of Biotechnology, Saveetha School of Engineering, Saveetha Institute of Medical and Technical Science (SIMATS), Chennai 602105, India; 2 School of Chemical Engineering, Yeungnam University, Gyeongsan 38541, Korea; 3 Department of Chemistry, Indian Institute of Technology, Roorkee 247667, India; 4 School of Bio-Sciences and Technology, Vellore Institute of Technology, Vellore 632014, India; 5 Department of Medical Biotechnology, Yeungnam University, Gyeongsan 38541, Korea; 6 Department of Chemistry, College of Natural Sciences, Kebri Dehar University, Korahe Zone, Somali Region, Kebri Dehar 3060, Ethiopia; 7 Research Institute of Cell Culture, Yeungnam University, Gyeongsan 38541, Korea

**Keywords:** cardiovascular disease, nano herb formulation, H9C2 cell line, apoptosis, ROS scavenging

## Abstract

Despite many efforts over the last few decades, cardiac-based drug delivery systems are experiencing major problems, such as the effective delivery of the precise amount of a drug. In the current study, an effort has been made to prepare a nano-herbformulation (NHF) to overcome the major problem of conventional intervention. Copper oxide-based NHF was prepared using plant extract of *Alternanthera sessilis* and characterized using physicochemical techniques such as Transmission electron microscopy (TEM), X-ray powder diffraction (XRD), Dynamic light scattering (DLS), UV-Vis spectroscopy, and Fourier-transform infrared spectroscopy (FTIR). TEM analysis revealed that spherical NHF obtained of size 20–50 nm. In addition, XRD and FTIR confirmed the presence of phytochemicals with biological properties over the surface of copper oxide-based NHF. It was demonstrated that dose-dependent antiapoptotic activity was shown against DOX-induced cardiomyocytes, where ROS levels were significantly reduced to 0.29% from 37.99%. The results of the flow cytometry analysis using PI and Annexin staining further confirmed the antiapoptotic activity of NHF against DOX-induced cardiomyocytes by ROS scavenging. Thus, NHF might be used for cardiovascular disease treatment.

## 1. Introduction

Nano-biotechnology combines nanotechnology with biotechnology to improve products and create new technologies. We manufactured several nanoparticle materials, including pigments, cosmetics, and biomedical devices. Nanomaterials such as copper oxide nanoparticles have made possible advances in pharmaceuticals, food packaging, and catalyst manufacturing [1,2]. There are several methods available for synthesizing nanoparticles [3]. To prevent agglomeration, we use toxic chemicals as reducing or stabilizing agents. CuO nanoparticles synthesized by chemical routes are also toxic to acute and chronic aquatic organisms [4]. Nano herb formulation contains a variety of active ingredients. Invade the targets of cells involved in developing various diseases [5]. The reason is that many of these nano-herbal compounds combine metal with many other bioactive compounds. The presence analysis of numerous chemical components to detect these presents a challenge for the chemist and pharmacist. These are due to the presence of the metal compound and the variety of herbal compounds. The present works were approached based on the Siddha principle.

Furthermore, researchers focus on developing a more reliable and green method of synthesizing nanoparticles with various herbal formulations acting against cardiovascular disease. Using optimized CuO nanoparticles with *Nelumbo nucifera* (leaf extract), *Spharanthus indicus* (Leaf extract), *Azardica indica* (flower extract), *Magnolia champaca* extract, this simple process provides nanoparticles of better physical and optical properties [6]. The search for traditional medicine novels and medicines continues [7]. Since paranoia has not been studied in any significant detail, it offers unique opportunities. Doxorubicin is a drug that can cause serious health problems and harm in many children and the elderly. The doxorubicin-induced cardiotoxicity is also. Patterns of proteomic signatures are used in serum analysis from rat models to detect anthracycline and anthracenedione-induced cardiotoxicity [8]. Although doxorubicin is present in the heart at relatively small concentrations, myocardial cells are susceptible to pharmacological effects [9]. Some enzymes are inhibited by doxorubicin, such as Na-K ATPase and myosin ATPase [10]. The biomarker, considered acute toxicity with nanoparticles, can be designed and harvested to absorb, enrich and multiply [11]. Doxorubicin (DOX) mediated cardiac toxicity involves alterations in the phosphate pool energy, disturbances in myocardial adrenergic signaling, and changes in endothelin-1 [12]. Doxycycline, a well-established tetracycline antibiotic and antimicrobial agent with minor side effects, even after long-term usage [13], can be employed as a probable novel therapeutic agent for cardiac failure.

All parts of *N. nucifera* have many medicinal uses. The leaf, rhizome, seed, and flower are traditionally used for the treatment of pectoralgia dysentery, cough, fever, pharyngoplasty, smallpox, spermatorrhoea, epistaxis, hyperdipsia, haematemesis, haemoptysis, haematuria, metrorrhagia, leucoderma, hyperlipidaemia, cholera, hepatopathy and has great medicinal properties as antidiarrheal, anti-obesity and hypocholesterolemic analgesic, hypolipidemic activity [14]. *Sphaeranthus indicus Linn*. is known as Koṭṭaikkarantai in Tamil. It is an important medicinal plant for treating nervous depression skin diseases, laxative, anthelmintic, antibiotics, styptic gastric disorders, glandular swelling, and analgesic, antifungal and diuretic properties [15]. *A. indica* flower is reviewed to be a strong antioxidant agent; it plays an essential role in preventing cancer progression and development, is antimicrobial, and has a major role in dentistry, inflammation, hepatoprotective effects, wound healing properties, antidiabetic, antimalarial, antiphrotoxicity, and neuroprotective [16]. *M. champaca* flower extract is used as a diaphoretic, purgative, diuretic, expectorant, cardiotonic, digestive, stomachic, carminative, stimulant, antipyretic and astringent [17]. 

In the field of Siddha medicine, natural remedies are used as medicine. They include herbs, metals, minerals, hydro chemicals, animal products, and arsenic. These are mainly used for pharmaceutical products [18]. It is used in biomedical medicine to treat many diseases, especially heart disease and cancer [19]. In the nanoparticles, copper chendhooram is a drug made of metals and minerals. These copper centaurs are said to retain their energy for 75 years. They are separated and combined with specific plant juices by nanotechnology, filtration or extraction, and sublimation or calculation or burning or frying or subjecting them to the process of exposure to insolation until the characteristic reddening of the product takes place.

Cardiomyoblast cells derived from mice are the H9C2 cell line. These similarities to different cardiomyocytes and have cardiac contractility and functional properties [20]. Heart disease is associated with a high level of ROS production [21]. This leads to oxidative stress, which causes significant damage to these ROS. Heart cells damage the oxidative system, leading to further apoptosis [22].

Nanotechnology has tremendous potential in treating and diagnosing genetic diseases/disorders with its medical implications as nanomedicine. It has revolutionary treatment with nanoparticles being directly incorporated into the genome to either treat/suppress/delete the disease-causing gene and thus cure the ailment with a high success rate commonly known as “target drug delivery” system [23], biosensors [24], cosmetic industry [25], and therapeutics [26]. 

## 2. Materials and Method

### 2.1. Collection and Identification of Plants

Our nano herbomineral formulation is synthesized using floral species such as *Alternanthera sessilis*, *Azadirachta indica*, *Sphaeranthus indicus*, *Nelumbo nucifera*, and *Michelia champaca*. Fresh leaves of *Sphaeranthus indicus*, *Alternanthera sessilis*, and *Azadirachta indica*, free from diseases, were collected from the Vellore institute of technology. *Nelumbo nucifera* and *Michelia champaca* were collected from Arulmigu Arunachaleswarar Temple, Thiruvannamalai, Tamilnadu, India.

### 2.2. Optimization and Formulation of Nano Herbomineral in Modified Siddha Approach

**Figure 1:**
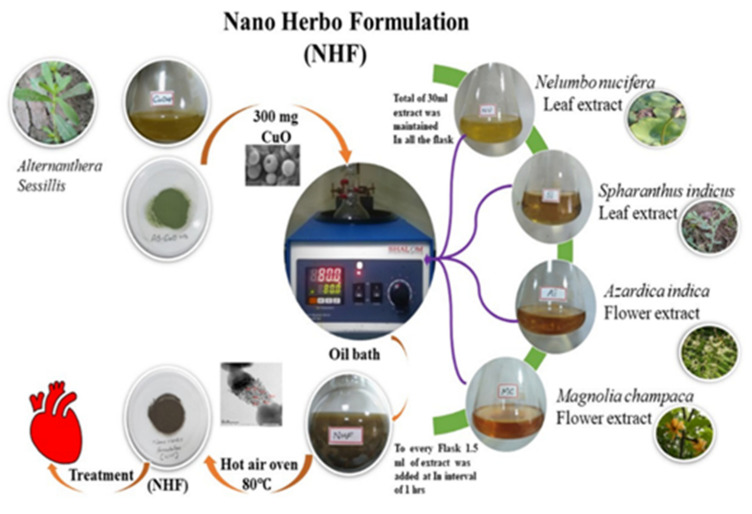
Schematic representation of Nano Herboformulation (NHF).

The CuONP required for our herboformulation was biosynthesized by a green method in *Alternanthera sessilis* and optimized using response surface methodology (RSM) [27]. RSM is an optimization approach using statistical analysis to optimize the parameters and determine the optimal conditions for obtaining nano-sized particles. Testing the existing drugs available in the market, followed by understanding the prime relationship based on polynomial mathematics and representing the results at optimum levels, are the main goals of this optimization method, as shown in Figure 1.

Initially, plant extract of *Alternanthera sessilis* and cupric chloride was used to synthesize CuONP (to eliminate the toxicity), which was later optimized to a size of 10 nm. Our formulation is entirely inspired by the prevailing Siddha medicine formulation in which they take pure copper along with the extract of other plants and synthesize the particles for around 32 days to eliminate its toxicity and achieve efficiency. However, here in our formulation methodology, we tried to reduce the synthesis time by opting for suitable technology and following the same flow for formulation. After the optimization of CuONP in a powdered concentration of 300 mg, it is placed in an oil bath. Parallelly 30 mL of another essential extract (i.e.) *Nelumbo nucifera* (leaf extract), *Spharanthus indicus* (Leaf extract), *Azardica indica* (flower extract), and *Mangolia champaca* (flower extract) were prepared. A time interval of 1 h 1.5 mL of the essential extract was added consecutively to the CuO particles placed in the oil bath, ensuring room temperature and rotation. After adding all the extract, the setup is left to formulate for 24 h. After the incubation of 24 h, the vial contents had filtered using a 0.22 μm cellulosic membrane filter, and obtained NHF was converted to powder form with the help of a hot air oven. Moreover, the entrapment efficiency (EE) of phytochemicals in NHF was calculated based on the following Equation (1)

(1) $$\%\;Entrapment\;efficiency=\frac{Total\;phytochemical-Free\;phytochemical}{Total\;phytochemical}\times 100$$

where UV-Vis measurements at 435 nm were used to assess content Vs (Standard Graph) 

### 2.3. Physiochemical and Compound Identification

Gas Chromatography-Mass Spectrometry/Mass Spectrometry was used to analyze NHF samples following liquid-liquid extraction with hexane and ethanol followed by ethanol. The extract was analyzed with UV-Vis DRS, X-Ray diffraction, FTIR, SEM, HRTEM, and DLS to determine its size, shape, and morphology. Elemental analysis of NHF was performed through the EDAX method. A different part of the plant was used to synthesize the nano herboformulation.

### 2.4. In-Vitro Drug Release Study

The cumulative phytochemical release from NHF formulation was performed. The NHF formulation at the concentration of 100.0 µg /mL in a dialysis bag taken in a beaker having the required pH buffer solution. The dialysis bag containing the NHF formulation was kept inside the buffer solution at 37 °C, and samples of the buffer were collected at regular intervals. The amount of drug release was measured by measuring the absorbance of the collected external buffer at 435 nm. The cumulative phytochemical release from NHF was determined by the following Equation (2)

(2) $$Phytochemical\;release\;\left(\%\right)=\frac{Phytochemical\;Released\;}{Total\;Phytochemical}\times 100$$

Furthermore, the kinetics of phytochemical release from NHF have been understood using the drug release dynamics using zero order, first order, and Korsmeyer-Peppas Model.

Zero-order is an independent kinetics model where the drug release rate is independent of its concentration and given by the Equation (3) as follows

(3) $$Q_{t}=Q_{0}-K_{0}t$$

where $Q_{0}$, is the initial amount of drug, $Q_{t}$ is the cumulative amount of drug released at a time “t”, $K_{0}$ is the zero-order rate constant, and t is the time.

Zero-order release kinetics is given by the graph plotted between the cumulative percentage of drugs released ($\frac{Q_{t}}{Q_{0}}$) versus time.

First order kinetics system describes that the drug release rate depends on its concentration and is given by the Equation (4) as follows

(4) $$log\;Q_{t}{}_{}=log\;Q_{0}+\frac{K_{1}}{2.303}\;$$

where $Q_{0}\;\mathrm{the}$ initial amount of the drug is, $Q_{t}$ is the cumulative amount of drug released at a time “t”, $K_{1}$ is the first order rate constant, and t is the time? The rate constant is given by plotting as log cumulative percentage drug remaining versus time where the slop is equal to $\frac{K_{1}}{2.303}$.

The Korsmeyer-Peppas kinetics Model is given by the Equation (5). As follows:

(5) $$F=\frac{M_{t}}{M_{\infty }}=K_{m}t^{n}\;$$

where the drug fraction released is denoted as F at a time ‘t’, M_t_ is the amount of drug released at a time ‘t’, M is the total amount of drug, K_m_ is the kinetic constant, and n is the diffusion or release exponent. The kinetic constant ‘K_m_’ and n was given by plotting the graph of log (Mt/M) versus log t. The value of *n* indicates the type of diffusion to be either fickian diffusion (*n* = 0.45), fickian diffusion (0.45 < *n* < 0.89), or case-2 relaxation (*n* = 0.89).

### 2.5. Cell Culture and Assessment of Cell Viability

In Dulbecco’s Modified Eagles Medium, H9C2 myocardial cells were purchased from National Centre for Cell Sciences, Pune, India. Under humidified 5% CO_2_ atmosphere at 37 °C, the cells at density 1 × 10^4^ counts were grown in 10% FBS, 1% penicillin-streptomycin antibiotic solution. MTT assay was used to assess cell viability. To reduce toxicity, we stimulated H9C2 myocardial cells with doxorubicin. NHF at different concentrations was used to reduce the toxicity of myocardial cells. A 96-well cell culture plate was filled with NHF with doxorubicin (5, 10, 20, 30, 40, and 50 µg/mL) for 30 min. The cells were incubated with various NHF Cells concentrations and then set at 37 °C in the dark for 4 h with MTT. After discarding the supernatant, 100 mL DMSO was added to the plates, dissolving the formazan crystals caused by MTT’s interaction with the live cells. Microplate readers were used to measuring the absorbance at 570 nm. 

### 2.6. Apoptosis and Cell Death Estimation

Following the manufacturer’s instructions, FITC Annexin V/Dead Cell Apoptosis Kit was used to analyze apoptosis. Following trypsinization, cells were harvested, maintained at 1 × 10^6^ counts, and suspended in Annexin binding buffer containing FITC Annexin and PI for 20 min in the dark. After that, the cells were suspended in a buffer containing propidium iodide for 5 min. An FC500 flow cytometer (Beckman Coulter, Indianapolis, IN, USA.) was used to acquire data for 10,000 events using three cell suspensions (5, 10, and 20 µg/mL).

### 2.7. Intracellular ROS Generation Estimation Using Flow Cytometry

Monitoring intracellular ROS was accomplished using dichloro-dihydro-fluorescein diacetate fluorescent probes. After 24 h of incubation in 6-well plates maintained at 1 × 10^6^ counts, cells were treated with NHF with doxorubicin (5, 10, or 20 µg/mL) and incubated for another 24 h. Incubation with DCFH-DA at 37 °C for about 30 min, followed by rinsing with fresh DMEM, was followed by analysis using a flow cytometer after cells were rinsed three times with fresh DMEM. Results were expressed as fold changes in fluorescence intensity based on a minimum of 10,000 events per sample. A comparison was made between the results and the control value.

### 2.8. Statistical Analysis

The experiments were run in triplicate, and the values were analyzed using the ANOVA statistical method. The results were statistically significant when the *p*-value was <0.05 and was expressed as the mean ± standard error.

## 3. Results

### 3.1. UV-Vis Spectra Studies

**Figure 2:**
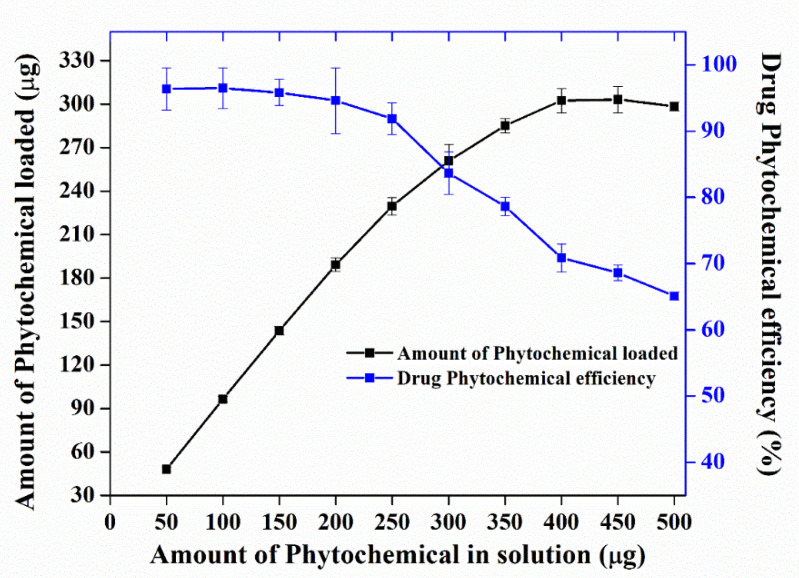
Drug loading capacity and efficiency of nano herboformulations.

**Figure 3:**
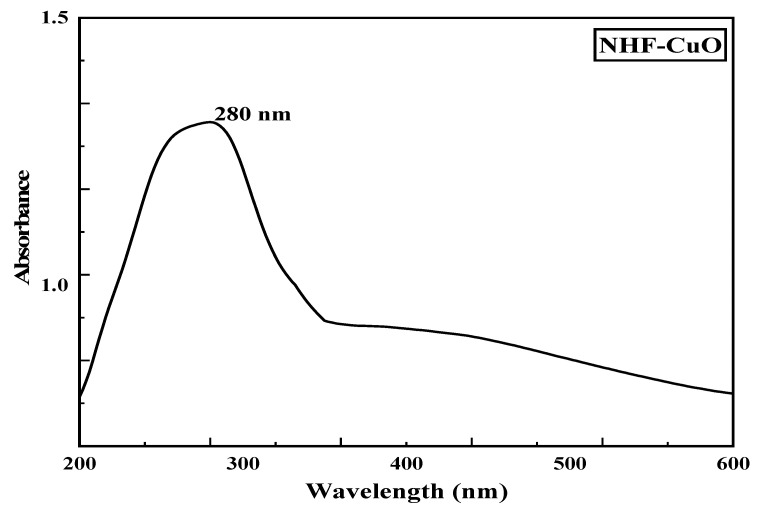
UV-Vis spectra of Nano Herboformulation.

Nano Herboformulation (NHF) was prepared as per the protocol described above, where the capacity of phytochemical in NHF was investigated and found to be 305.51 µg/mg with a loading efficiency of around 61.58% in Figure 2. UV-Vis spectroscopy is a widely recognized methodology for studying powder-suspended nanoparticles [28]. At room temperature, the UV-Visible absorption spectra of the green synthesized nanoformulations are presented in Figure 3. As a result of surface plasmon resonance (SPR), the nanoformulations show high absorption below 350 nm and a well-defined absorbance peak at around 280 nm. Copper oxide nanoparticles synthesized from various plant precursors also showed a similar result [29].

### 3.2. XRD and FTIR Analysis of Nano Herboformulation

**Figure 4:**
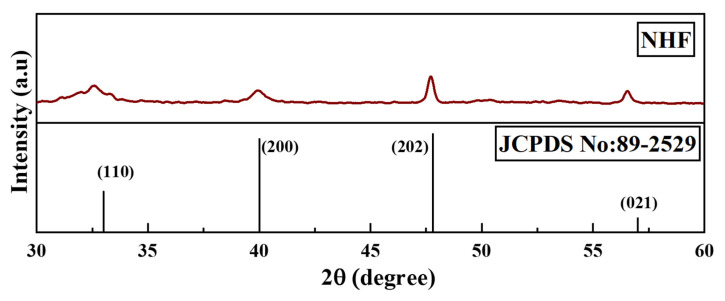
X-ray diffraction pattern of Nano Herboformulation.

XRD technique was used to analyze the microcrystalline structure of nano herboformulation. Characteristic XRD peaks of the nanoformulations were observed at 32.7, 40.2, 46.4, and 57.3, correspondings to 110, 200, 202, and 021 planes, respectively, as shown in Figure 4. This observation indicates the formation of monoclinic CuO. The NP system is seen as a typical result. Their sharp peaks confirm CuO NP. Reveals the crystalline nature of the further confirmation (JCPDS card number 89-2529). The XRD crystal structure provides the lattice parameters, nature of the phase, and crystal grain size [30]. Our Sharer equation is used to determine how broadening the most extreme peak of an XRD measurement is for a model. The average size of the nano herboformulation was 7.70 nm.

**Figure 5:**
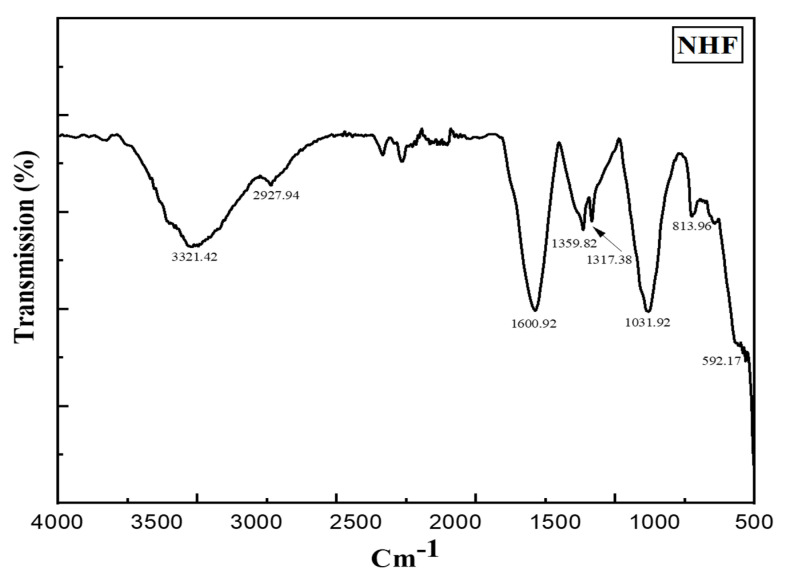
FT-IR spectrum of Nano Herboformulation (1317.38 cm^−1^peak corresponding C-H deformation vibration).

In FTIR analysis, we were able to classify nano-sized substances by the composition of nano-herboformulation. Figure 5 shows FTIR spectra of nano herboformulation. Nano herboformulation FTIR spectra show Cu-O vibrations at frequencies less than 700 cm^−1^ [31]. Small peaks (592.17 cm^−1^), O-H stretching vibration (3321.42 cm^−1^), frequency-independent (2927.94 cm^−1^), vibration resulting from C=C stretching (1600.92 cm^−1^), O-H deformation vibration (1359.82 cm^−1^), and bands corresponding to the characteristic frequency of inorganic ions are observed in CuO NPs [32]. Alkane, aromatic, ketone compounds, carboxylic acids, and aromatic nitro compounds are attributed to C-H deformation vibration (1317.38 cm^−1^) and CH_3_ rocking vibration (1031.92 cm^−1^). Deflation of the capping caused a comparatively high peak in CuO NPs due to C-O stretching [33].

### 3.3. Size and Shape Determination of Nano Herboformulation

**Figure 6:**
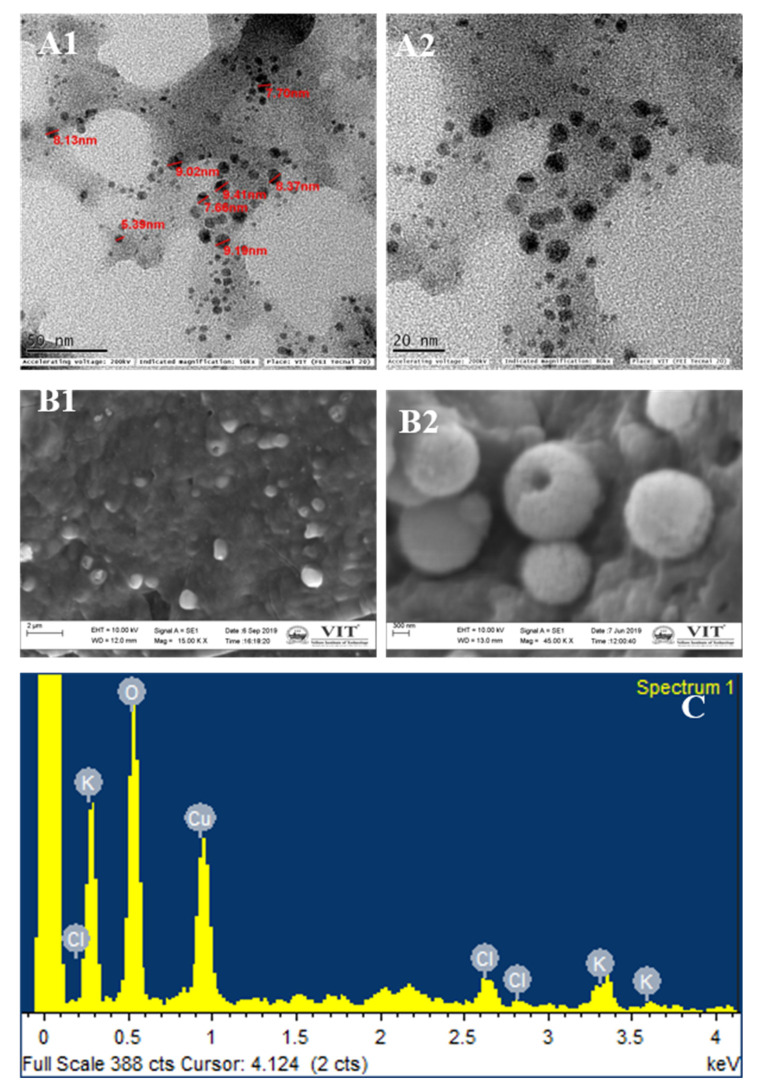
Electron micrographs of Nano Herboformulation. (**A1**,**A2**) TEM analysis. (**B1**,**B2**) SEM analysis, (**C**) EDX analysis of Nano Herboformulation, where O—48.41%, Cl—5.77%, K—8.29%, Cu—37.23%.

The standard physicochemical characterization used for integrated nano-herb formation. As shown in Figure 6A of TEM and Figure 6B of SEM, spherical particles of size ranging between 5–10 nm were identified. The amount of nano-herb formulation differs depending on the solvent medium as plant juices are attached to them. Thus, the cytotoxicity effect and the extent of its medium [34]. SEM showed that the nano herboformulation’s nano shape is approximately 300 nm Figure 5B. The shape of nanoparticles in the nano herboformulation plays a significant role in influencing the distinctive process of nanoparticle interaction with living patterns [35]. In in vivo tests, nanoparticles less than 10–20 nm are rapidly distributed over neuronal administration between all organs and tissues, while larger nanoparticles ranging from 50–250 nm are found in the blood, liver, spleen, and heart [36].

EDX microanalysis is used to create maps of various chemical components. This map is generated using software that evaluates the required element concentration at each imaging stage (X-ray spectra) in scanning mode. EDX analysis confirmed copper oxide nanoparticles and trivial quantities of Cl and K Figure 6C. The occurrence of copper and oxygen confirmed the formation of copper oxide nanoparticles in the reaction system. However, minor amounts of other reserves were found, which may be the molecule used in the analysis.

### 3.4. Zeta Potential and Particle Size of Nano Herboformulation

**Figure 7:**
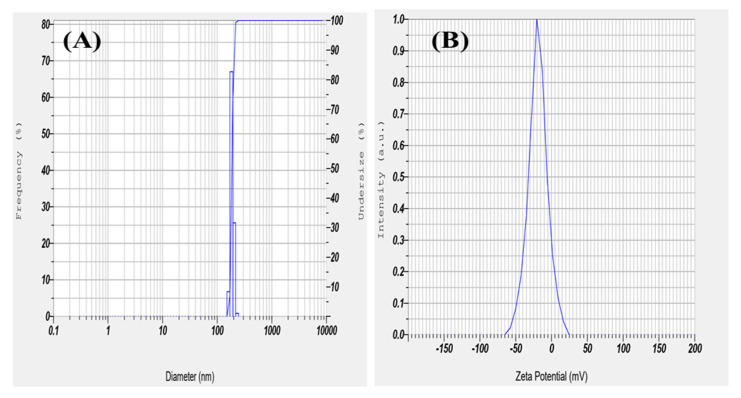
(**A**) Dynamic light scattering and (**B**) Zeta Potential of Herboformulation.

The size and stability of nano herboformulation are essential for medicinal application. To assess the stability of nanofluids, Zeta observes the electrophoretic behavior of the potential analysis fluid [37]. As shown in Figure 7B, a high Zeta potential value represents the stability of nano herboformulations in an aqueous medium. A zeta potential value of −20.58 mV shows colloidal stability. The nanoparticle size and colloidal stability were determined using DLS [38]. The hydrodynamic diameter of the nano herboformulation was determined to be 12.9 nm Figure 7A.

### 3.5. GC-MS Analysis of Nano Herboformulation

**Figure 8:**
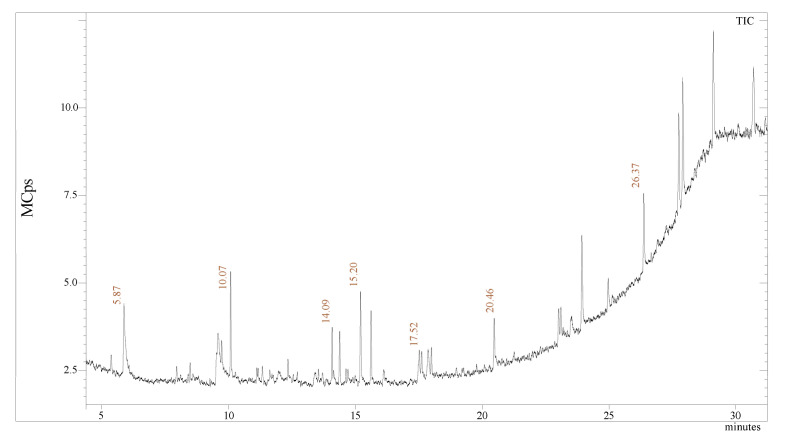
GC-MS analysis: chromatogram of the NHF.

The presence of a compound responsible for a different type of plant reacts with copper oxide. The Gas chromatography-mass spectrometry of nano herboformulation showed 13 peaks that were identified after comparing the mass spectra with Library used NIST Version-2011, indicating the presence of 13 phytocompounds. Figure 8 represents the formulation’s possible components (based on the NIST Library). The compounds are listed in Table 1. along with their peak area and RT molecular weight. Represents compound from 1H-Inden-1-one,2,3-dihydro 5,6-dimethody-3-methyl (RT 10.07), performed experiments revealed that 10e has an anti-β amyloid effect can reduce ROS, LDH, and MDA also a positive posing impact on TAC [39]. Similarly, 9-octadecenoic acid resists doxorubicin and cytarabine-induced oxidative stress. Oleic acid (RT), as reported in our present findings (RT 20.46) compound had more favorable effects similar effects on non-HDL-cholesterol concentrations and fasting LDL-cholesterol in comparison to hexadecenoic acid [40].

### 3.6. In-Vitro Phytochemical Release Study of NHF Formulation

**Figure 9:**
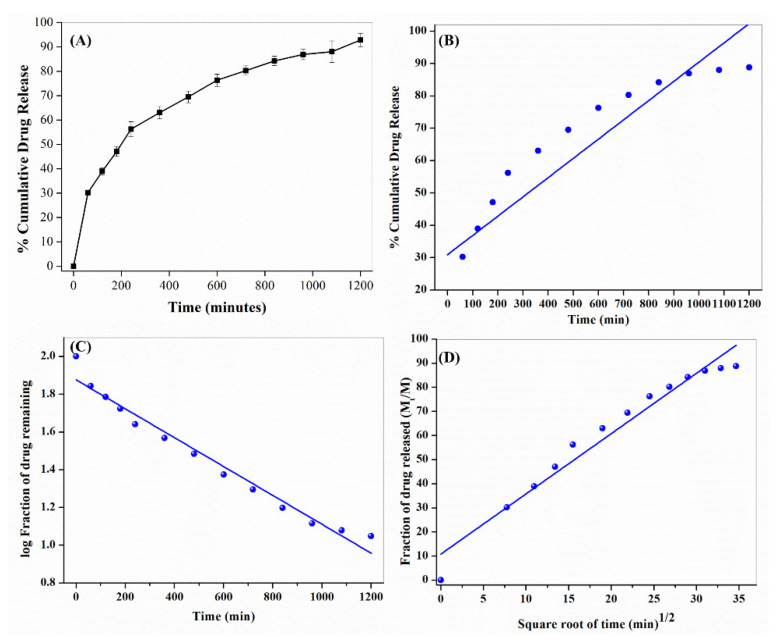
(**A**) Phytochemical release study from NHF. Kinetics of study phytochemical release study from NHF (**B**) Zero-Order, (**C**) First-Order & (**D**) Korsmeyer–Peppas models.

We have also studied the *in-vitro* phytochemical release study of *NHF formulation*, nearly 94.2 % of the *phytochemical* release (Figure 9A). The *phytochemical* release kinetics data from *NHF formulation* were also fitted to the different kinetics models (Table 2). It was found best to follow the Korsmeyer-Peppas model, and hence, the release of the drug must be mediated by diffusion mechanism, especially non-fickian diffusion (0.45 < *n* < 0.89) type of transport mechanism (Figure 9B–D). 

### 3.7. Cell Viability, Oxidative Stress, and Apoptosis in Doxorubicin-Induced h9c2 Cell Line

The study of different cell biology techniques, the analysis of reactive oxygen species, the analysis of apoptosis, and the identification of morphological changes. Using H9C2 cells to induce doxorubicin treatment, we explored the role of nano herbal formulations in reducing cardiotoxicity. We then examined the protective capacity of the nano herboformulations in myocardial cells from H9C2. 

#### 3.7.1. Dose-Response Curve Using MTT Assay—Cell Viability Assay

**Figure 10:**
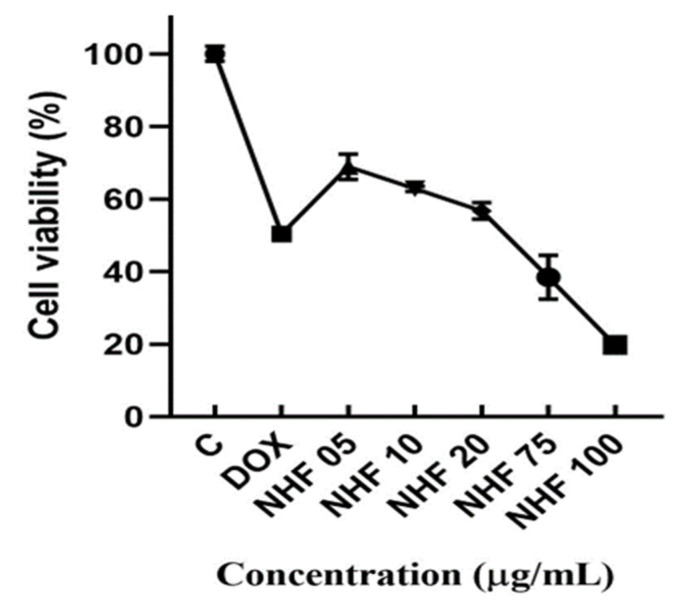
MTT analysis for NHF against DOX-induced H9c2 cell lines. All the data are expressed as mean ± SD (n = 3). (*p* < 0.05).

H9C2 cardiomyoblast cells viability to various concentrations of doxorubicin-induced nano herboformulation was studied for 24 h. Dose-dependent cytotoxicity was observed with doxorubicin treatment (Figure 10). DOX-induced cells showed stable cytotoxicity after NHF treatment and higher mitochondrial oxidative pressure after NHF treatment. An MTT assay can distinguish membrane lysis from non-membrane lysis by changing MTT reductase activity.

Cell viability was found to be increased in NHF-treated cells in a concentration-dependent manner Figure 10. Cells were treated with (1 µM) of doxorubicin to induce cytotoxicity in H9C2 cells and with NHF at different concentrations (5, 10, 20, 30, 40, and 50 µg/mL) to revert the induced toxicity. As shown in the graph, we found 5, 10, and 20 µg/mL concentrations to have high % cell viability; thus, we chose this concentration for further in-vitro studies. NHF of higher concentrations (5, 10, 20 µg/mL). Based on earlier studies, doxorubicin and levosimendan increased cell endurance and prevented oxidative stress-induced cell death in rodent hepatocytes [41]. Evaluation of the cytotoxic effects due to various nanoparticles for cells H9C2, A549, HEK293, and MCF-7 shows that fundamentally distinct nanoparticles impose unique biological and toxic effects [42]. Cells treated with NHF for 24 h showed no significant toxic effects. Co-treatment using a similar cocktail formula did not show a significant improvement in cell viability, which is also noticeable compared to free dox (*p* > 0.05) [43]. The experiments were carried out in triplicates, and the calculated values are mean ± SD. On the contrary, NHF at (5, 10, 20 µg/mL) dosage increased the viability of H9C2 cells to near normal.

#### 3.7.2. NHF in Oxidative Stress Induced by DOX-Treatment in H9c2 Cells

**Figure 11:**
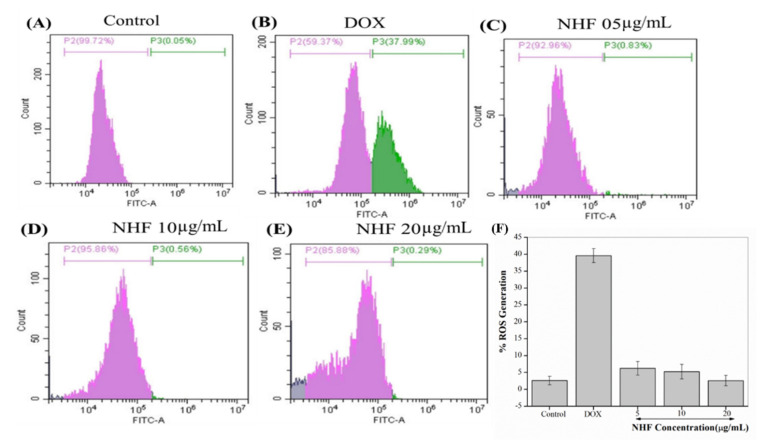
NHF-induced oxidative stress in H9C2 cardiomyocytes. (**A**) Control (**B**) Dox-induced ROS (**C**) NHF 5 µg/mL (**D**) NHF 10 µg/mL (**E**) NHF 20 µg/mL (**F**) complementary representation of ROS.

Purine nucleotide acts as an intermediate to DOX metabolize quinone by flavoproteins. Doxorubicin damaged approximately 37.99% of the cells. After DOX treatment of NHF cell damage, DOX-induced H9C2 cells showed a low level of (0.83%, 0.56%, and 0.29%) (Figure 11 C-E) compared to control cells. Figure 11 A-B shows how NHF affects Dox-induced ROS production within cells. Here we have treated doxorubicin-induced toxicity with NHF at 5, 10, and 20 µg/mL Concentrations. 

The formation of the DOX-iron complex stimulates ROS generation. The results in hydrogen peroxide (H_2_O_2_) and superoxide ions [44]. Moreover, H_2_O_2_ is degraded by low molecular weight clay irons into common reaction hydroxyl radical [45]. Hyper generation of ROS leads to antioxidant pressure, which is activated by DOX and stimulates the apoptotic signaling layer in cardiomyocytes [46]. Figure 11 F complementary graph of ROS. As shown in the image, all treated groups showed reduced ROS levels compared to control cells. The ROS levels were drastically reduced in NHF treatment.

#### 3.7.3. NHF in Apoptosis Induced by DOX-Treatment in H9c2 Cells

**Figure 12:**
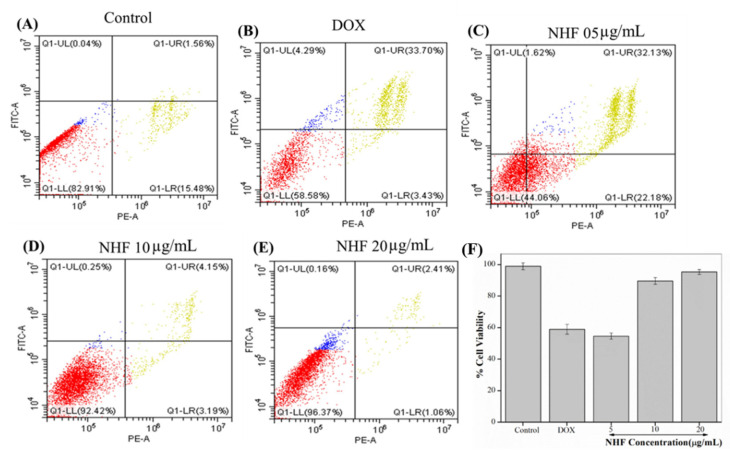
Analyses of flow cytometry using PI and Annexin staining of H9C2 cells. (**A**) Control (**B**) Dox-induced apoptosis (**C**) NHF 5 µg/mL (**D**) NHF 10 µg/mL (**E**) NHF 20 µg/mL (**F**) Complementary representation of apoptosis.

Here we have seen apoptosis assay for NHF. As shown in Figure 12A, the Control and doxorubicin-treated cell are the same as before. Figure 12C–E showed cells treated with 5, 10, and 20 µg/mL Figure 12C concentration showed late apoptosis cells. Figure 12D showed fewer late apoptotic cells treated with 10 µg/mL. Figure 12E shows 20 µg/mL concentration when we found reduced apoptosis cells compared to all other treatments, and Figure 12F complementary graph of apoptosis.

The results of NHF with doxorubicin-induced antioxidant pressure are a significant factor in toxicity. The potential role of oxidative stress in mediation with apoptosis induction and DNA damage has been documented [47]. At the end of these studies, our results demonstrate that NHF oxidative stress, DNA damage, and apoptosis triggers are lower than in previous reports [48].

## 4. Conclusions

Cardiovascular disease poses a significant threat to society as it holds the highest mortality rate. Here, we have encountered the formation of nano herbal has shown good progress in cardiac function. From this study, it seems possible to shunt CVD harmlessly and healthily using Nano herbo-formulation with reduced toxicity and increased therapeutic support. The overall analysis of the relationship between nano herboformulation revealed by the transformation of value in H9C2 and zebrafish is given with doxorubicin, confirming the reduced toxicity while using nano herboformulation. It provides the exact measures and the role of ROS and the apoptotic analysis in H9C2. Overall, this gives a clear output for further expanding the nano herboformulation in the treatment of CVD. In future studies, the most common preclinical model, the nano herboformulations showing the highest potential in this in vitro model, can be further assessed in mice. It is possible to reduce research time and costs associated with high numbers of mice using this approach. The addition of this animal model would strengthen and improve the approval process for nano herboformulation-based cardiac and cancer treatments.

## Figures and Tables

**Table 1:** Compound identification from Nano herboformulation.

S. No	RT.	Name of the Compound	Molecular Formula	Molecular Weight	Peak Area %
1.	5.87	Ethanol, 2-phenoxy-	C_8_H_10_O_2_	138	14.46
2.	9.58	Cyclobarbital	C_12_H_16_N_2_O_3_	236	12.35
3.	9.73	2-Allyl-3,6-dimethoxybenzyl alcohol	C_12_H_16_O_3_	208	4.71
4.	10.07	1H-Inden-1-one, 2,3-dihydro-5,6- dimethoxy-3-methyl-	C_12_H_14_O_3_	206	13.29
5.	11.23	1,2-Benzisothiazol-3-amine tbdms	C_13_H_20_N_2_SSi	264	1.27
6.	12.35	2-Myristynoyl-glycinamide	C_16_H_28_N_2_O_2_	280	2.36
7.	14.09	Z, E-2,13-Octadecadien-1-ol	C_18_H_34_O	266	5.99
8.	14.38	Phen-1,4-diol, 2,3-dimethyl-5- Trifluoromethyl-	C_9_H_9_F_3_O_2_	206	7.28
9.	15.20	Dodecanoic acid, 10-methyl-, methyl ester	C_14_H_28_O_2_	228	11.81
10.	17.52	6-epi-shyobunol	C_15_H_26_O	222	6.11
11.	17.99	d-Mannitol, 1-decylsulfonyl-	C_16_H_34_O_7_S	370	3.30
12.	20.46	Oleic Acid	C_18_H_34_O_2_	282	6.21
13.	26.37	Di-n-decylsulfone	C_20_H_42_O_2_S	346	10.85

**Table 2:** Drug release data were obtained from various kinetic models.

Model	R^2^	SST
Zero-order kinetic model	0.8562	0.094
First-order kinetic model	0.8745	0.0354
Korsmeyer–Peppas kinetic model	0.9764	1.43

## Data Availability

Not applicable.
